# Variations in morbidity and mortality based on esophagectomy approach and leak status: Insights from a statewide quality collaborative

**DOI:** 10.1016/j.xjon.2025.11.010

**Published:** 2025-11-19

**Authors:** Jenny Bui, Donald Chang, Katelyn R. Ward, Mary Hollenbeck, Andrew M. Popoff, Thomas J. Watson, Kiran Lagisetty, Shelly Lall, Kumari Adams, Geoffrey Lam, Rishindra M. Reddy

**Affiliations:** a Department of Surgery, Section of Thoracic Surgery, University of Michigan, Ann Arbor, Mich; b Department of Surgery, Henry Ford, Detroit, Mich; c Department of Surgery, Corewell Health East William Beaumont University Hospital, Royal Oak, Mich; d Michigan Society of Thoracic and Cardiovascular Surgeons Quality Collaborative, Ann Arbor, Mich; e Division of Thoracic Surgery, Henry Ford Health, Detroit, Mich; f Division of Thoracic Surgery, Corewell Health East William Beaumont University Hospital, Royal Oak, Mich; g Division of Thoracic Surgery, Munson Medical Center, Transverse City, Mich; h Division of Thoracic Surgery, Trinity Health Ann Arbor Hospital, Ann Arbor, Mich; i Division of Cardiothoracic Surgery, Corewell Health West, Meijer Heart Center, Grand Rapids, Mich

**Keywords:** anastomotic leak, esophagectomy, transhiatal, transthoracic

## Abstract

**Objective:**

Anastomotic leak remains a major source of morbidity after esophagectomy. Cervical anastomoses have been associated with higher leak rates compared with intrathoracic anastomoses; however, cervical leaks may have a less severe clinical impact. The aim was to determine whether the patterns of leak occurrence and severity varied by surgical approach among institutions at a regional level.

**Methods:**

We retrospectively reviewed data for patients who underwent open or minimally invasive transhiatal esophagectomy or transthoracic esophagectomy for esophageal cancer between January 2015 and March 2023 at 13 centers. Patients were stratified by procedure type and leak rates, and postoperative complications were examined. Logistic regression assessed associations among the surgical technique, leak rates, and postoperative complications.

**Results:**

A total of 1230 patients undergoing esophagectomies were reviewed: 500 (41%) with open transhiatal esophagectomy, 135 (11%) with open transthoracic esophagectomy, 258 (21%) with minimally invasive transhiatal esophagectomy, and 337 (27%) with minimally invasive transthoracic esophagectomy. Leak rates ranged from 14% to 23%, with the highest rate observed in minimally invasive transhiatal esophagectomy and the lowest in open transthoracic esophagectomy (*P = .*081). Among those with leaks, open transthoracic esophagectomy had the longest length of stay (25 days, interquartile range, 12-33) compared with transhiatal esophagectomy groups (12-14 days, *P = .*003). Pneumonia (42%, *P < .*001), empyema requiring intervention (21%, *P = .*004), and sepsis (26%, *P = .*027) were significantly higher in the open transthoracic esophagectomy group compared with other groups. In-hospital mortality after a leak was highest in open transthoracic esophagectomy (16%) compared with 3% in open transhiatal esophagectomy and 1% in minimally invasive transhiatal esophagectomy and transthoracic esophagectomy groups (*P = .*025).

**Conclusions:**

Although leak rates are higher with transhiatal esophagectomy, open transthoracic esophagectomy is associated with greater morbidity and mortality after leak.

## Graphical Abstract

**Figure:**
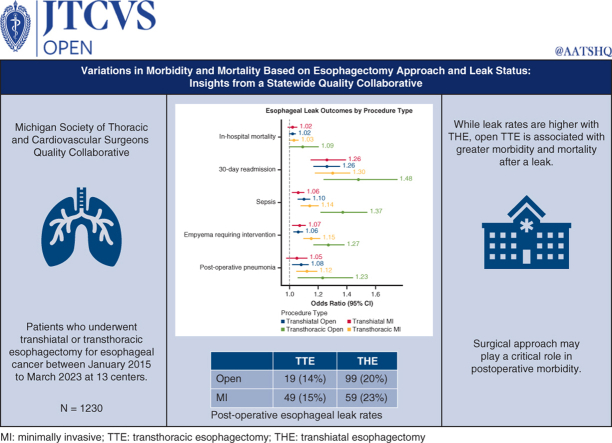

**Figure:**
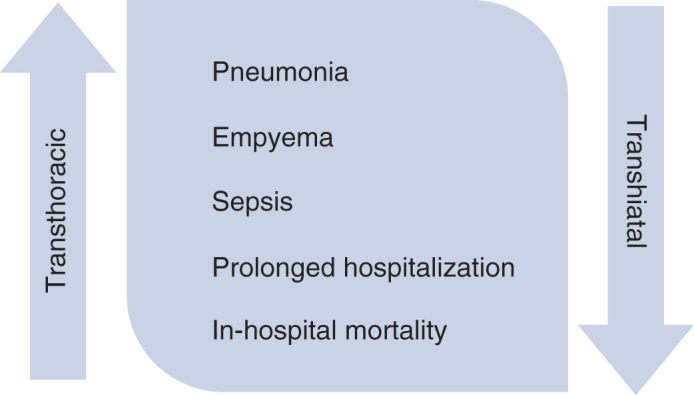
Increased morbidity and mortality with open TTE compared with THE.

**Central Message**

Although leak rates are higher with THE, open TTE is associated with greater morbidity and mortality after a leak.

**Perspective**

This is a contemporary, real-world analysis focusing on the relationship between the surgical approach and the incidence, severity, and clinical impact of anastomotic leaks after esophagectomy for esophageal cancer. Open THE was associated with worse clinical outcomes after a leak, suggesting that surgical approach plays a critical role in postoperative morbidity.

Curative treatment for esophageal cancer often involves surgical resection, complemented by neoadjuvant or adjuvant chemotherapy, radiotherapy, and now immunotherapy.^1^, ^2^, ^3^ The management of esophageal cancer has evolved with various surgical techniques, including the transthoracic or Ivor Lewis (2-stage esophagectomy with abdominal gastric mobilization, transthoracic esophageal dissection, and intrathoracic anastomosis) and transhiatal (abdominal gastric mobilization, transhiatal dissection of the lower esophagus, and cervical anastomosis) esophagectomies.^4^ Despite these advancements, there remains debate about the optimal surgical approach to maximize oncologic results and minimize risks.

Anastomotic leaks are still one of the most critical complications after esophageal cancer resections, contributing significantly to postoperative morbidity and mortality. Significant clinical consequences include higher rates of postoperative complications, prolonged hospital stays, and increased healthcare costs.^5^ These leaks may result in severe infections such as mediastinitis or sepsis, leading to the need for reinterventions such as drainage, stent placement, or additional surgeries.^5^ Patients who experience anastomotic leaks also tend to have a higher risk of mortality, with some studies reporting mortality rates between 3% and 12%.^6^^,^^7^ Beyond immediate complications, the presence of an anastomotic leak can delay or prevent the initiation of adjuvant therapies such as chemotherapy or radiation, potentially impacting long-term survival. Leaks also may contribute to the development of anastomotic strictures, often requiring repeat endoscopic dilations.^8^ In addition to the physical outcomes, anastomotic leaks can lead to poorer nutritional status and the need for enteral or parenteral feeding.^5^ Thus, preventing anastomotic leaks is crucial to improving both short- and long-term clinical outcomes for patients with esophageal cancer. Cervical anastomoses are associated with higher rates of leakage compared with intrathoracic anastomoses, although the clinical impact of cervical leaks is generally less severe.^9^

Within the Michigan Society of Thoracic and Cardiovascular Surgeons Quality Collaborative (MSTCVS-QC), there is a concerted effort to evaluate current surgical practices and outcomes across institutions. By leveraging shared data, the collaborative aims to identify practice variation and its impact on patient outcomes, with the ultimate goal of driving improvements in the quality and safety of esophageal cancer care. As such, we sought to review current leak rates and associated outcomes to identify opportunities for targeted improvement across participating hospitals. The aim was to determine whether the patterns of leak occurrence and severity varied by surgical approach among institutions at a regional level.

## Material and Methods

This study was deemed exempt by the University of Michigan Institutional Review Boards as a quality improvement study (IRB #HUM00213629; 04/07/2022).

### Data Sources and Study Sample

We retrospectively reviewed the Society of Thoracic Surgery General Thoracic Surgery Database (STS-GTSD) data from the MSTCVS-QC from 13 hospitals and queried patients undergoing transhiatal esophagectomy (THE) or transthoracic esophagectomy (TTE), open or minimally invasive (MI), for esophageal cancer between January 2015 and March 2023. Informed consent was waived due to the retrospective nature of the study and minimal risk to participants. During this period, 3-incision esophagectomy procedures were excluded because of the low number of cases. All records were submitted to the STS-GTSD using the major procedure Data Collection Form version 2.3 (January 1, 2015 to July 1, 2019), version 2.41 (July 2, 2019, to June 30, 2021), and version 5.21 (July 1, 2021, to March 31, 2023). Clinical staging was based on endoscopic ultrasound or computed tomography per STS-GTSD guidelines. Intraoperative conversions were coded in the registry and analyzed based on the technique that was used to successfully complete the operation.

### Outcomes Definition

The primary outcome was the development of a postoperative anastomotic leak.

Anastomotic leak was defined in accordance with coding guidelines from the STS-GTSD. A postoperative leak was coded as “yes” if any of the following conditions were met: radiographic evidence of a leak on contrast esophagram (eg, barium swallow); clinical suspicion of a leak prompting an intervention such as percutaneous or operative drain placement, esophageal stent placement, administration of antibiotics for suspected or confirmed leak, or return to nothing by mouth status; or a return to the operating room for washout, drainage, or repair. Additionally, opening of a bedside incision for drainage or wound management, or explicit documentation by the operating or attending surgeon indicating the presence of a postoperative leak was sufficient to meet the definition.

Additional secondary outcomes included in-hospital postoperative complications, in-hospital mortality, 30 day-readmission, and 30-day mortality. The study used International Classification of Diseases, Ninth Revision, Clinical Modification procedure codes, and Current Procedural Terminology codes to identify the various surgical approaches, with robotic assistance for procedures specifically coded in the database. One in-hospital postoperative complication, empyema requiring intervention, was defined as an infected pleural space necessitating further antibiotic therapy or placement of additional chest tubes or drains. Diagnosis was confirmed via thoracentesis, with findings of frank pus or cloudy pleural fluid, leukocytosis, low pH (<7.2), low glucose (<60 mg/dL), elevated lactate dehydrogenase and protein, and the presence of infectious organisms.

### Statistical Analysis

Demographic data are reported as mean (SD) or median (interquartile range [IQR]) for continuous variables and frequencies (percentages) for categorical variables. Continuous data that followed a normal distribution were analyzed using 1-way analysis of variance, and non-normal distributed data were assessed using the Kruskal–Wallis test. For categorical data, comparisons were made using the Pearson chi-square test or Fisher exact test, as appropriate. Logistic regression using robust SEs was conducted to examine the relationship between the surgical technique and the likelihood of postoperative complications after an anastomotic leak. The covariates included age, sex, race, smoking status, number of medical comorbidities, clinical tumor stage, nodal involvement, receipt of preoperative chemotherapy or radiation, cancer histology, cancer location, and hospital volume. Because surgical approach and year were highly correlated in the dataset, we did not include year as a covariate in the primary multivariable models to minimize multicollinearity and avoid sparse cell bias. To explore temporal effects, a descriptive secondary analysis was performed stratified by 2 time periods (2014-2018 vs 2019-2024) to assess trends in leak rates over time. These analyses were intended to clarify temporal patterns but were not incorporated into the primary regression models. Additionally, given the regionalization of MI-THE to a small number of high-volume centers, a sensitivity analysis was conducted to assess whether this concentration biased our findings. The primary regression model was repeated after excluding MI-THE cases from the highest volume hospital. All *P* values were 2-tailed. Statistical analyses were performed using Stata software version 17 (StataCorp).

## Results

### Patient Characteristics

A total of 1230 patients undergoing esophagectomies were reviewed: 500 (41%) with open THE, 135 (11%) with open TTE, 258 (21%) with MI-THE, and 337 (27%) with MI-TTE. The median age was similar across groups, ranging from 65 to 67 years (Table 1). A majority of patients were male (77%-87%, *P = .*018) and White (89%-95%, *P = .*014). The predominant histologic subtype was adenocarcinoma (86%-92%). Preoperative treatment patterns were also similar, with 79% to 82% of patients receiving preoperative chemotherapy and 77% to 79% of patients receiving preoperative radiation therapy. Between 2014-2018 and 2019-2024, the proportion of MI procedures increased substantially—transthoracic from 19.9% to 28.1% and transhiatal from 14.1% to 22.6% (*P* < .001). Robotic assistance was more frequently used in MI-THE procedures (87%) compared with MI-TTE cases (62%) (*P < .*001). Median lymph nodes sampled ranged from 12 to 18, with MI-TTE yielding the highest nodal counts (18, IQR, 12-24) compared with open THE (12, IQR, 8-16) (*P < .*001). Baseline demographics are shown in Table 1.

**Table 1:** Patient demographics

Demographic	Transhiatal open N = 500	Ivor Lewis open N = 135	Transhiatal MI N = 258	Ivor Lewis MI N = 337	*P*
Age (median, IQR), y	66 (59-72)	65 (59-71)	67 (59-72)	66 (59-71)	.501
Male gender (%)	424 (85%)	118 (87%)	198 (77%)	276 (82%)	**.018**
Racial demographics					**.014**
White (%)	473 (95%)	128 (95%)	242 (94%)	300 (89%)	
Black (%)	3 (1%)	2 (1%)	7 (3%)	15 (4%)	
Asian (%)	7 (1%)	1 (0.7%)	3 (1%)	1 (1%)	
Other (%)	11 (2%)	0 (0%)	4 (0.4%)	6 (2%)	
Smoking status (%)					**.001**
Never	92 (18%)	21 (16%)	34 (13%)	63 (19%)	
Former	341 (68%)	79 (59%)	179 (69%)	208 (62%)	
Current	38 (8%)	27 (20%)	21 (8%)	29 (9%)	
DM (%)	90 (18%)	22 (16%)	34 (13%)	47 (14%)	.906
HTN (%)	230 (46%)	58 (43%)	89 (34%)	138 (41%)	.559
CAD (%)	99 (20%)	25 (19%)	38 (15%)	59 (18%)	.384
CHF (%)	6 (1%)	7 (5%)	3 (1%)	6 (2%)	**.015**
CVA (%)	13 (3%)	4 (3%)	9 (3%)	14 (4%)	.653
Preoperative chemotherapy (%)	401 (80%)	110 (81%)	203 (79%)	276 (82%)	.783
Preoperative radiation (%)	390 (78%)	104 (77%)	201 (78%)	266 (79%)	.972
Robotic technique (%)	--	--	224 (87%)	210 (62%)	**<.001**
Clinical stage (%)					.322
T1	73 (15%)	19 (14%)	30 (12%)	32 (9%)	
T2	128 (26%)	27 (2%)	65 (25%)	73 (22%)	
T3	284 (57%)	81 (60%)	156 (60%)	222 (66%)	
T4	8 (2%)	3 (2%)	4 (2%)	4 (1%)	
Nodal involvement (%)	248 (50%)	65 (48%)	123 (48%)	167 (50%)	.949
Cancer histology (%)					
Adenocarcinoma	443 (89%)	121 (90%)	223 (86%)	309 (92%)	**.003**
Squamous cell	46 (9%)	5 (4%)	34 (13%)	16 (5%)	
Other	4 (1%)	0 (0%)	1 (0.4%)	5 (1%)	
Cancer location (%)					
Upper third	6 (1%)	2 (1%)	2 (0.8%)	1 (0.3%)	**.002**
Middle third	25 (5%)	1 ((0.7%)	17 (7%)	7 (2%)	
Lower third	359 (72%)	85 (63%)	185 (72%)	232 (69%)	
Gastroesophageal junction	110 (22%)	47 (35%)	54 (21%)	97 (29%)	
Total LNs sampled, median (IQR)	12 (8-16)	15 (9-24)	15 (9-18)	18 (12-24)	**<.001**

*IQR*, Interquartile range; *DM*, diabetes mellitus; *HTN*, hypertension; *CAD*, coronary artery disease; *CHF,* congestive heart failure; *CVA*, cerebrovascular accident; *LN,* lymph node.

*P*-values < .05 are labelled in bold.

### Overall Postoperative Outcomes by Procedure Type

The median length of stay (LOS) varied significantly across procedure groups (*P = .*001), with patients undergoing MI-THE having the shortest median LOS (8 days, IQR, 7-10) compared with 10 days (IQR, 8-14) in both open and MI-TTE and 9 days (IQR, 8-13) in open THE (Table 2). Postoperative esophageal leaks occurred in 14% to 23% of patients, with the highest rate observed in MI-THE (23%) and the lowest in open TTE (14%), although this did not reach statistical significance (*P = .*081). Temporal stratification showed that leak rates increased significantly over time in MI-ILE (*P = .*017) and open THE (*P* < .001), while remaining stable in the MI-THE and open ILE groups. Postoperative pneumonia rates differed significantly across groups (*P* < .001), with patients undergoing open TTE having the highest incidence (18%) compared with open THE (7%), MI-THE (6%), and MI-TTE (7%). Delayed conduit emptying was most common in MI-TTE (6%) and least common in MI-THE (1%) (*P* < .001). Sepsis rates were significantly different across groups, with open TTE having the highest rate (11%, *P* < .001). Readmission within 30 days was most common in open TTE (26%) and least common in MI-THE (14%, *P = .*025). In-hospital mortality differed significantly among groups with open TTE having the highest rate (5%), whereas open THE, MI-THE, and MI-TTE had in-hospital mortality rates of 1% (*P = .*011).

**Table 2:** Postoperative outcomes by procedure type

Outcomes	Transhiatal Open N = 500	Transhiatal MI N = 258	Ivor Lewis Open N = 135	Ivor Lewis MI N = 337	*P*
LOS, median (IQR), d	9 (8-13)	8 (7-10)	10 (8-14)	10 (8-14)	**.001**
Unplanned transfer to ICU (%)	42 (8%)	25 (10%)	15 (11%)	17 (5%)	.159
Esophagogastric leak (%)	99 (20%)	59 (23%)	19 (14%)	49 (15%)	.081
Postoperative arrythmia (%)	116 (23%)	66 (26%)	38 (28%)	61 (18%)	.119
Postoperative pneumonia (%)	35 (7%)	16 (6%)	24 (18%)	24 (7%)	**<.001**
Delayed conduit emptying (%)	13 (3%)	3 (1%)	4 (3%)	21 (6%)	**<.001**
Conduit necrosis (%)	4 (1%)	0 (0%)	0 (0%)	2 (1%)	.433
Empyema requiring intervention (%)	12 (2%)	6 (2%)	7 (5%)	12 (4%)	.135
Sepsis (%)	18 (4%)	6 (2%)	15 (11%)	14 (4%)	**<.001**
30-d readmission (%)	92 (18%)	37 (14%)	35 (26%)	59 (18%)	**.025**
In-hospital mortality (%)	6 (1%)	3 (1%)	7 (5%)	5 (1%)	**.011**
30-d mortality (%)	2 (0.2%)	1 (0.3%)	1 (1%)	3 (1%)	.777

*MI,* Minimally invasive; *LOS,* length of stay; *IQR*, interquartile range; *ICU,* intensive care unit.

*P*-values < .05 are labelled in bold.

### Postoperative Outcomes in Patients With Esophageal Leaks by Procedure Type

**Figure 1:**
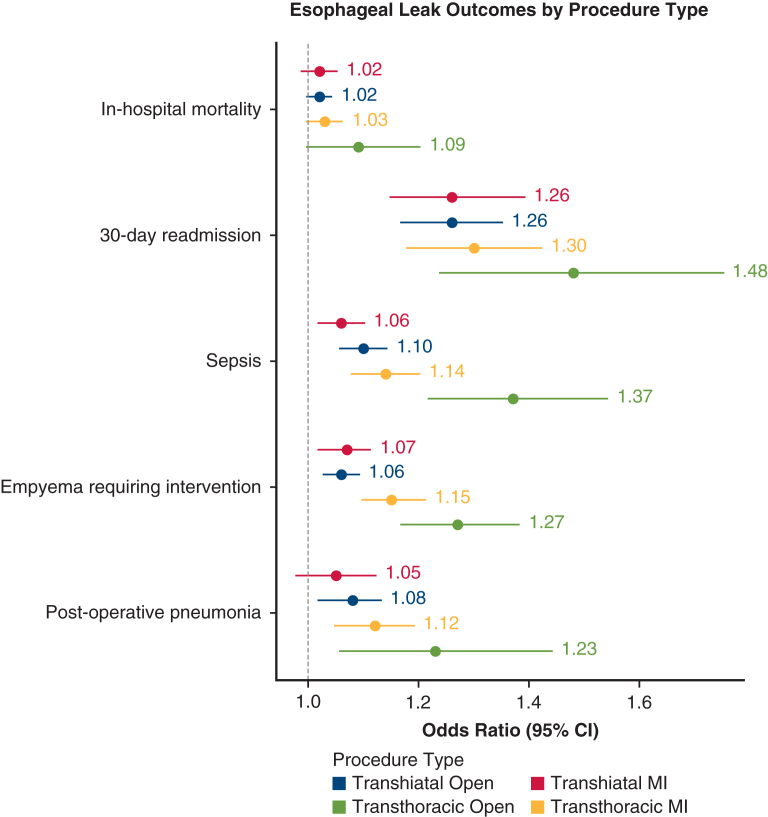
Odds of postoperative complications after an esophageal leak. *MI,* Minimally invasive.

Nineteen patients (14%) in the open TTE group, 49 patients (15%) in the MI-TTE group, 99 patients (20%) in the open THE group, and 59 patients (23%) in the MI-THE group developed a postoperative anastomotic leak. Among patients who developed a leak, LOS varied significantly across procedural groups (*P = .*003; Tables 3 and 4). Open TTE had the longest median LOS (25 days, IQR, 12-33), followed by MI-TTE (17 days, IQR, 13-31). In contrast, open THE and MI-THE had shorter LOS of 14 days (IQR, 11-25) and 12 days (IQR, 8-16), respectively (Tables 3 and 4). Postoperative pneumonia in patients with a leak occurred more frequently in open TTE (42%) compared with MI-TTE (16%), open THE (12%), and MI-THE (7%) (*P* < .001). Likewise, empyema requiring intervention was significantly more common in open TTE (21%) and MI-TTE (20%) compared with open THE (10%) and MI-THE (5%) (*P = .*004). Sepsis rates also differed significantly across groups, with the highest incidence in open TTE (26%, *P = .*027). In-hospital mortality was significantly higher in open TTE (16%) compared with 3% in open THE, 4% in MI-TTE, and 2% in MI-THE (*P = .*025). Patients with an esophageal leak after an open TTE had the highest likelihood of postoperative pneumonia (odds ratio [OR], 1.24, 95% CI, 1.06-1.44, *P = .*007), empyema requiring intervention (OR 1.27, 95% CI, 1.17-1.38, *P < .*005), sepsis (OR, 1.37, 95% CI, 1.23-1.54, *P < .*005), and 30-day readmission (OR, 1.48, 95% CI, 1.25-1.75, *P < .*005) (Figure 1). The direction and relative magnitude of associations remained consistent in sensitivity analyses, even when excluding the highest-volume MI-THE center, suggesting that the main findings were not driven solely by institutional outlier effects.

**Table 3:** Postoperative outcomes for patients with leaks by procedure type

Outcomes	Transhiatal open N = 99	Transhiatal MI N = 59	Transthoracic open N = 19	Transthoracic MI N = 49	*P*
LOS, median (IQR), d	14 (11-25)	12 (8-16)	25 (12-33)	17 (13-31)	**.003**
Transfer to ICU (%)	17 (17%)	11 (19%)	5 (26%)	9 (18%)	.719
Postoperative arrythmia (%)	28 (28%)	19 (32%)	8 (42%)	14 (29%)	.659
Postoperative pneumonia (%)	12 (12%)	4 (7%)	8 (42%)	8 (16%)	**<.001**
Delayed conduit emptying (%)	8 (7%)	1 (2%)	0 (0%)	3 (6%)	.200
Conduit necrosis (%)	2 (2%)	0 (0%)	0 (0%)	1 (2%)	.633
Empyema requiring intervention (%)	5 (5%)	4 (7%)	4 (21%)	11 (20%)	**.004**
Sepsis (%)	8 (8%)	3 (5%)	5 (26%)	8 (15%)	**.027**
30-d readmission (%)	37 (37%)	22 (34%)	11 (58%)	23 (53%)	.091
In-hospital mortality (%)	3 (3%)	1 (2%)	3 (16%)	2 (4%)	**.025**
30-d mortality (%)	0 (0%)	1 (2%)	0 (0%)	2 (4%)	.221

*MI,* Minimally invasive; *LOS,* length of stay; *IQR*, interquartile range; *ICU,* intensive care unit.

*P*-values < .05 are labelled in bold.

**Table 4:** Postoperative outcomes for patients without leaks by procedure type

Outcomes	Transhiatal open N = 401	Transhiatal MI N = 199	Transthoracic open N = 116	Transthoracic MI N = 228	*P*
LOS, median (IQR), d	9 (8-12)	7 (7-9)	9 (8-13)	9 (8-12)	**<.001**
Transfer to ICU (%)	25 (6%)	14 (7%)	10 (7%)	8 (3%)	.063
Postoperative arrythmia (%)	88 (22%)	47 (24%)	30 (26%)	47 (16%)	.091
Postoperative pneumonia (%)	23 (6%)	12 (6%)	16 (14%)	16 (6%)	**.013**
Delayed conduit emptying (%)	5 (1%)	2 (1%)	4 (4%)	18 (6%)	**<.001**
Conduit necrosis (%)	2 (0.5%)	0 (0%)	0 (0%)	1 (0.4%)	.681
Empyema requiring intervention (%)	7 (2%)	2 (1%)	4 (21%)	11 (20%)	.230
Sepsis (%)	10 (2%)	2 (2%)	10 (9%)	6 (2%)	**.001**
30-d readmission (%)	55 (14%)	15 (8%)	24 (21%)	36 (13%)	**.009**
In-hospital mortality (%)	3 (0.8%)	2 (1%)	4 (3%)	4 (1%)	.151
30-d mortality (%)	2 (0.5%)	0 (0%)	1 (0.9%)	1 (0.4%)	.672

*MI,* Minimally invasive; *LOS,* length of stay; *IQR*, interquartile range; *ICU,* intensive care unit.

*P*-values < .05 are labelled in bold.

## Discussion

This analysis retrospectively reviewed a statewide quality collaborative database from 13 participating institutions with varying practice types (academic vs community, low-volume vs high-volume center) across the state of Michigan. To support ongoing quality improvements efforts, the aim was to evaluate the incidence and clinical consequences of postoperative anastomotic leaks of patients undergoing esophagectomy for cancer based on surgical approach.

Consistent with prior studies, this study reaffirms that THE is associated with a higher leak rate compared with TTE, with observed leak rates of 20% to 23% for THE versus 14% to 15% for TTE. Leak rates in this cohort were somewhat higher than those reported in certain single-institution series, which likely reflects the nature of real-world data collected across a statewide collaborative, encompassing diverse practice settings and varying levels of surgeon experience. Between 2014-2018 and 2019-2024, the proportion of MI procedures increased substantially—transthoracic from 19.9% to 28.1% and transhiatal from 14.1% to 22.6%. The rise in leak rates over time likely reflects a statewide learning curve as MI techniques became more widely adopted across centers, introducing variability in experience and case complexity. Additionally, improved reporting and postoperative surveillance may have contributed to greater leak detection in the more recent era. However, Hagens and colleagues^9^ reported leak rates of 14% to 31% for cervical anastomoses compared with a 3% to 17% leak rate for intrathoracic anastomoses. This difference may be explained by the longer distance required for vascular perfusion to reach a cervical anastomosis, potentially compromising healing compared with the shorter intrathoracic anastomosis in TTE.^10^ In addition, increased anastomotic tension is a major risk factor contributing to higher leak rates in this group.^11^ However, although intrathoracic anastomoses may leak less frequently, our findings demonstrate that their clinical consequences are often more severe. Patients who developed leaks after open TTE had the highest rates of pneumonia, empyema, sepsis, prolonged hospital stays, and in-hospital mortality. By contrast, MI-TTE was associated with lower morbidity, shorter LOS, and complication rates more comparable to THE, suggesting that the excess risk is largely due to open TTE rather than the TTE approach itself. TTE places the esophagogastric anastomosis within the thoracic cavity, specifically in the posterior mediastinum, a space where leaks will contaminate the pleural and mediastinal spaces even with adequate drainage.^4^^,^^5^ As a result, leaks in this area can rapidly lead to mediastinitis, empyema, and systemic sepsis. An open surgical approach may further exacerbate the issue by increasing the overall inflammatory response and pain impairing respiratory function postoperatively, thus contributing to a higher incidence of pulmonary complications such as pneumonia.^12^

In contrast, patients with leaks after THE experienced shorter lengths of stay, fewer infectious complications, and lower mortality rates, suggesting a less severe clinical course despite the higher incidence of leaks. Despite a potentially higher incidence of leaks, these leaks are more frequently contained and amenable to conservative management because the leak can drain from the cervical wound.^13^, ^14^, ^15^ Interestingly, MI-THE was associated with the shortest LOS and lowest complication rates among patients with leaks, which may reflect the benefits of MI access or be due to specific surgeon experience. Supporting this, a retrospective comparison between conventional TTE and THE and MI esophagectomies found that patients who had a MI esophagectomy had shorter operative times, less blood loss, fewer transfusions, and shortened intensive care unit and hospital courses.^16^^,^^17^

These observations support the notion that cervical leaks may be more frequent but less morbid, whereas intrathoracic leaks, particularly after open TTE, can lead to more serious complications due to their proximity to the mediastinum and higher risk of pleural contamination. The elevated rates of sepsis and empyema in patients receiving TTE reinforce this concern.

This study did not demonstrate an increased risk of mortality in patients with an anastomotic leak compared with those without leaks, although the study may be underpowered to demonstrate this. Other prior studies reported no difference in short- or long-term survival based on leak status.^9^^,^^18^ The literature on survival after anastomotic leak is mixed: Some studies have shown a significant association between leaks and decreased survival, whereas others have not.^18^, ^19^, ^20^, ^21^ This variation is likely due to heterogeneity in leak severity, location, and differences in diagnosis and management. For example, Markar and colleagues^22^ found that severe anastomotic leaks were associated with a 28% increased risk of long-term mortality, whereas less severe leaks had no survival difference compared with patients without leaks. Our data set did not assess the severity of leaks, which could have helped understand the impact in a more granular level.

**Figure 2:**
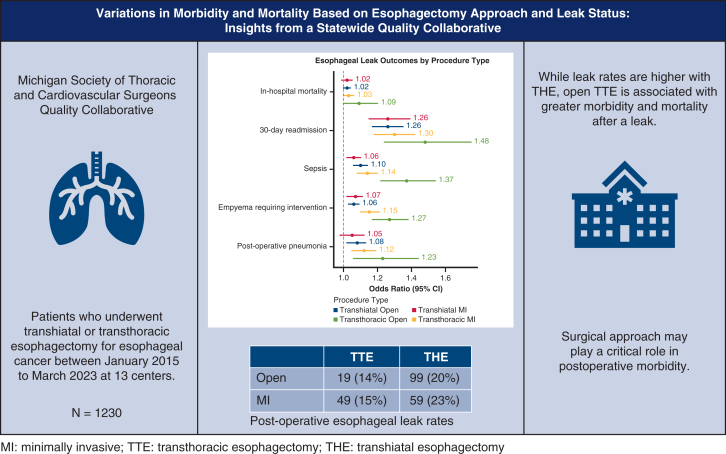
Graphical Abstract- Eight year data from a regional quality collaborative suggests higher leak rates after transhiatal esophagectomy, and higher morbidity and mortality after an open transthoracic esophagectomy.

This study contributes to the ongoing quality improvement discussions regarding the optimal surgical approach for esophageal cancer. The data suggest that MI techniques, including both MI-THE and MI-TTE, may offer favorable short-term outcomes even in the event of a leak, whereas the increased morbidity and mortality are largely driven by open TTE (Figure 2).

### Study Limitations

This study has several limitations. As a retrospective analysis, it is dependent on the accuracy of STS database coding, which may vary across institutions despite ongoing audits by the MSTCVS-QC Coordinating Center. The collaborative's use of the STS-GTSD and its associated submission guidelines also limit the availability of long-term outcomes, such as 90-day mortality, which could not be assessed. The database also does not reliably capture whether procedures were performed in a hybrid fashion, and therefore outcomes could not be stratified by hybrid versus total MI approaches. Operating room times are not reliably comparable across institutions in this dataset, because they are recorded differently depending on local protocols and workflows. For this reason, operating times were not analyzed. In addition, the inclusive definition of anastomotic leak may have captured a broad range of severities, from subclinical radiographic findings to clinically significant leaks requiring reintervention. Variation in imaging practices, diagnostic thresholds, and institutional coding rigor may have influenced the reported incidence. Surgeon experience and learning curve could not be assessed directly, but outcomes at lower-volume hospitals may have been influenced by differences in experience and annual case volume. Finally, the study population was limited to cardiothoracic surgeons participating in the STS-GTSD and this quality collaborative, which may introduce selection bias. Despite these limitations, our findings provide important insights into the relationship between the surgical approach and the incidence, severity, and clinical impact of anastomotic leaks after esophagectomy for cancer.

## Conclusions

In this multicenter analysis of esophagectomy for cancer, the type of surgical approach was closely associated with both the incidence and clinical consequences of anastomotic leaks. Although THE was associated with a higher leak rate, open TTE resulted in the most severe postoperative complications, including higher rates of pneumonia, empyema, sepsis, prolonged hospitalization, and in-hospital mortality. These findings suggest that the excess morbidity and mortality traditionally due to transthoracic approaches are largely driven by the presence of a thoracotomy, particularly when a leak occurs. MI approaches, including both MI-THE and MI-TTE, were associated with more favorable outcomes even in the presence of leaks, highlighting that avoidance of a thoracotomy may be key to minimizing the risks associated with esophagectomy.

## Conflict of Interest Statement

A.M.P.: Consultant for Intuitive, Oxford Performance Materials, and Premier Inc. K.L.: AtriCure (Grants). R.M.R.: Intuitive Surgical (teaching site/consultant), AtriCure and On Target Laboratories (Advisory Board and Grants), Medtronic and Genentech (Advisory Board), BMS (Speaker), Trinity Health (Consultant). Support for the MSTCVS-QC is provided by Blue Cross Blue Shield of Michigan and Blue Care Network as part of the BCBSM Value Partnerships program. Although Blue Cross Blue Shield of Michigan and MSTCVS-QC work collaboratively, the opinions, beliefs, and viewpoints expressed by the authors do not necessarily reflect the opinions, beliefs, and viewpoints of BCBSM or any of its employees. All other authors reported no conflicts of interest.

The *Journal* policy requires editors and reviewers to disclose conflicts of interest and to decline handling or reviewing manuscripts for which they may have a conflict of interest. The editors and reviewers of this article have no conflicts of interest.
